# Valence, arousal, and task effects in emotional prosody processing

**DOI:** 10.3389/fpsyg.2013.00345

**Published:** 2013-06-21

**Authors:** Silke Paulmann, Martin Bleichner, Sonja A. Kotz

**Affiliations:** ^1^Department of Psychology and Centre for Brain Science, University of EssexColchester, UK; ^2^Department of Neurology and Neurosurgery, Rudolf Magnus Institute of Neuroscience, University Medical Center UtrechtUtrecht, Netherlands; ^3^Department of Neuropsychology, Max Planck Institute for Human Cognitive and Brain SciencesLeipzig, Germany; ^4^School of Psychological Sciences, University of ManchesterManchester, UK

**Keywords:** P200, LPC, ERPs, arousal, task demands, emotion, prosody

## Abstract

Previous research suggests that emotional prosody processing is a highly rapid and complex process. In particular, it has been shown that different basic emotions can be differentiated in an early event-related brain potential (ERP) component, the P200. Often, the P200 is followed by later long lasting ERPs such as the late positive complex. The current experiment set out to explore in how far emotionality and arousal can modulate these previously reported ERP components. In addition, we also investigated the influence of task demands (implicit vs. explicit evaluation of stimuli). Participants listened to pseudo-sentences (sentences with no lexical content) spoken in six different emotions or in a neutral tone of voice while they either rated the arousal level of the speaker or their own arousal level. Results confirm that different emotional intonations can first be differentiated in the P200 component, reflecting a first emotional encoding of the stimulus possibly including a valence tagging process. A marginal significant arousal effect was also found in this time-window with high arousing stimuli eliciting a stronger P200 than low arousing stimuli. The P200 component was followed by a long lasting positive ERP between 400 and 750 ms. In this late time-window, both emotion and arousal effects were found. No effects of task were observed in either time-window. Taken together, results suggest that emotion relevant details are robustly decoded during early processing and late processing stages while arousal information is only reliably taken into consideration at a later stage of processing.

## Introduction

There is a recent increase in studies informing about the complexity and diversity of how the brain processes emotional information from the voice. Much progress has been made in depicting which brain structures are implied during emotional prosody processing, that is the variation of acoustic cues such as fundamental frequency (F_0_), amplitude (or intensity), timing, and voice quality (energy distribution) during speech (see e.g., Kotz and Paulmann, 2011 for recent review). In addition, electrophysiological studies have investigated the time-course or speed with which emotional prosodic information is processed to ensure appropriate social behavior (e.g., Pihan et al., 1997; Schirmer et al., 2002, 2005b; Schirmer and Kotz, 2003; Bostanov and Kotchoubey, 2004; Wambacq et al., 2004; Kotz and Paulmann, 2007; Paulmann and Kotz, 2008; Paulmann and Pell, 2010). However, although most researchers would agree that emotional information as conveyed by the voice (or other non-verbal channels such as face or body posture) can be described in a two-dimensional space, that is, with regard to valence (pleasant – unpleasant) and arousal (sometimes referred to as activation: high – low; see e.g., Feldman-Barrett et al., 2007 for review of emotion theories), most electrophysiological research on vocal emotion processing has concentrated on exploring when *emotional* or *valence* attributes are processed, thereby ignoring the possible contribution of *arousal* during emotional prosody processing. Thus, the present investigation aims to start filling this gap in the literature by studying how and when these two dimensions impact on emotional prosody processing.

Event-related brain potentials (ERPs) have been widely used to define the temporal processes involved in emotional prosody processing. For instance, early studies on vocal emotion processing have focused on assessing when stimuli of different valences can be distinguished from one another (e.g., Wambacq and Jerger, 2004; Schirmer et al., 2005a). Later studies have explored when language stimuli expressing so-called basic emotions (anger, fear, disgust, sadness, surprise, happiness) can be differentiated from neutral stimuli and/or each other (e.g., Paulmann and Kotz, 2008; Paulmann et al., 2011). Generally speaking, ERP findings support the notion that valence information is detected and analyzed rapidly (within the first 200 ms after stimulus encounter) from prosody (e.g., Schirmer et al., 2005a, 2013; Paulmann and Kotz, 2008; Garrido-Vásquez et al., in press), irrespective of speaker voice (Paulmann and Kotz, 2008; Paulmann et al., 2008b), and even when information is not task-relevant (Wambacq et al., 2004; Kotz and Paulmann, 2007), or when it is processed pre-attentively (Schirmer et al., 2005a). Under attentive processing conditions, the process of rapid emotional salience detection has repeatedly been linked to the P200 component, a fronto-centrally distributed positivity reaching its peak approx. 200 ms after stimulus onset.

While the early P200 component is assumed to reflect enhanced attention to emotional stimuli so that they can be preferentially processed if need be, (concurrent) later ERP components are often linked to more in depth processing mechanisms (e.g., meaning evaluation, access to memory representation). Specifically, late emotional prosody effects have been observed in several late ERP components including the P300 (e.g., Wambacq and Jerger, 2004), N300 (e.g., Bostanov and Kotchoubey, 2004), N400 (e.g., Schirmer et al., 2002, 2005a; Schirmer and Kotz, 2003; Paulmann and Pell, 2010), and a late positive complex (LPC; Kanske and Kotz, 2007; Schirmer et al., 2013), depending on stimuli, tasks, and experimental designs used. Thus, a growing body of literature suggests that emotion signaling features such as valence or even emotional category knowledge are rapidly extracted and analyzed during emotional prosody processing. However, next to nothing is known about additional emotion relevant parameters that could potentially influence this early evaluation process. Specifically, so-called circumplex models of emotion propose that both valence and arousal dimensions are crucial when describing how someone feels (e.g., Feldman-Barrett and Russell, 1998; Feldman-Barrett, 2006), that is both dimensions should modulate how emotions are perceived from speech.

While previous electrophysiological research on *vocal* emotional language processing has either ignored the dimension of arousal altogether or has tried to control for arousal by keeping activation attributes of stimuli similar, ERP research on *visual* emotional language processing has already started to explore the (combined) influence of valence and arousal on processing affective word or sentence stimuli. For instance, Hinojosa et al. (2009) presented positive prime-target word pairs which were either congruent or incongruent with regard to their arousal level. Participants were instructed to identify whether the target word was of either high or low (relaxing) arousal. The authors report reduced LPC amplitudes for high-arousal congruent target words when compared to high-arousal incongruent target words. This priming effect occurred between 450 and 550 ms after target word onset and was interpreted to reflect reduced attentional resources needed to process highly arousing stimuli when preceded by stimuli of the same arousal level (Hinojosa et al., 2009). Similarly, Bayer et al. (2010) report a short negativity between 330 and 430 ms after stimulus onset for sentences containing negative high-arousal target words when compared to sentences with negative low-arousal target words while participants engaged in a semantic judgment task (does the target word fit the preceding context). Combined, their results are in line with the view that arousal relevant details about word stimuli are processed at a rather “late” (cognitive) processing stage compared to valence or emotion relevant details, which have been reported to be processed in earlier processing stages (e.g., Gianotti et al., 2008). In other words, findings from studies exploring visual emotional language processing suggest that arousal influences allocation of attentional resources and later sustained stimulus evaluation processes while valence or emotion attributes of stimuli can impact early, initial evaluation of stimuli which ensures that potentially relevant stimuli are preferentially processed over irrelevant (c.f., Hinojosa et al., 2009). This view has also received support from studies using non-language emotional stimuli such as pictures (see Olofsson et al., 2008 for review). It should not go unmentioned that there is also some sparse evidence that arousal of language stimuli can modulate early ERP components: Hofmann et al. (2009) reported that high-arousal negative words elicit an increased early negative ERP between 80 and 120 ms after word onset in contrast to neutral and low-arousal negative words when participants performed a lexical decision task. This greater ERP negativity was linked to an early effect of arousal on lexical access processing. Specifically, the authors interpreted the ERP amplitude differences between high-arousal negative and neutral words to reflect early facilitative lexical access for arousing negative stimuli suggesting an early influence of arousal on affective word processing. The same effect, however, was not found for positive word stimuli, that is, the general influence of arousal on early emotional word processing mechanisms remains to be further investigated. Given that different studies applied different tasks, it can also not be excluded that varying task demands (explicit emotional/arousal focus, implicit emotional/arousal focus) could partly account for the equivocal time-course findings in the literature.

We are unaware of electrophysiological studies exploring the influence of arousal and valence on emotional prosody processing in a combined experimental design. Thus, the current study tested the influence of arousal and valence on both early (P200) and late (LPC) ERP components by using pseudo-sentence stimuli intoned in six distinct emotional tones (anger, disgust, fear, sadness, surprise, happiness). Given that emotions can be expressed with either high or low arousal (e.g., one can say “stop” in a calm but firm, or in a shriek voice; both times expressing anger), stimuli were also grouped according to arousal level of the speakers, who intoned the sentences so that each emotional category contained sentences that were rated as either low or high arousing. To test for the influence of task focus, half of the participants were asked to rate the arousal level of the speaker who intoned the sentence they had just heard, while the other half was asked to rate how aroused they felt after listening to the sentence. Thus, task demands (e.g., processing effort) are comparable as in both instances, participants made use of a nine-point Likert scale; however, task focus is different in that one group focused on the arousal level of the presented stimuli (explicit task), while the other group focused on their own arousal level (implicit task). In view of previous findings from emotional visual language processing (see above), we hypothesized that stimuli expressing different emotions (and valences) would elicit differently modulated P200 amplitudes (rapid emotional salience detection) as well as differently modulated LPC amplitudes (sustained emotional evaluation). In contrast, arousal effects should only modulate ERPs in a later time-window (LPC) if true that emotional relevant attributes (e.g., saliency, category knowledge) are processed before arousal relevant attributes (i.e., determine the calmness/excitation of a stimulus). However, in light of findings which suggest that arousal effects might be modulated by task focus (explicit vs. implicit), a potential influence of arousal on early processing mechanisms could not be completely ruled out.

## Materials and methods

### Participants

Forty right-handed native speakers of German (21 female, mean age: 25 years, range: 20–30 years) participated in the study. Data from one participant had to be excluded due to excessive muscle movements during the electroencephalogram (EEG) recording. None of the participants reported any hearing impairments, and all had normal or corrected-to-normal vision. Participants gave their written informed consent and the experiment was approved by the Ethics Committee of the Max Planck Institute (CBS, Leipzig). All participants were compensated financially for their participation.

### Stimulus material

Emotional portrayals were elicited from two native German actors (one male, one female). Recordings were made with a digital camcorder connected to a high quality clip on microphone. During the recording session, actors produced pseudo-sentences, that is sentences which contain prosodic information but no semantic content, belonging to one of six basic emotional (happiness, pleasant surprise, anger, disgust, fear, sadness), or a neutral category. Stimuli were phonotactically and morpho-syntactically legal in German (example: Mon set die Brelle nogeferst and ingerafen). We presented a total of 360 emotional sentences (30 sentences per emotional category, each spoken by a male and a female speaker) and 50 different neutral filler sentences (again, each spoken by both speakers). Each neutral sentence was repeated three to four times (per speaker) throughout the experiment to ensure that an equal amount of emotional and neutral stimuli were presented (360 sentences each). Given that neutral sentences lack the dimension of arousal (high vs. low), they were not included in the analysis and solely served as filler material. All sentences were rated for their emotional tone of voice by 24 participants (12 female, none of the raters participated in the present study) in a forced-choice paradigm. The mean percentage agreement for the sentences selected for the present study was: 90.66% for anger, 68.18% for disgust, 60.78% for fear, 68.17% for sadness, 57.55% for happiness, 54.34% for pleasant surprise, and the mean percentage correct for neutral was 90.09%. Further rating details can be found in Pell et al. (2009). See Table 1 for results of acoustical analyses of stimuli. Comparable to the majority of previous studies exploring emotional prosody processing, stimuli were not artificially matched for amplitude, pitch, or tempo across emotional categories to ensure natural-like material. In addition, arousal ratings for stimuli were obtained from participants of the current study who rated materials for arousal level of the speaker (explicit task, see below). For each emotional category, sentences were grouped according to the arousal level as expressed by the speaker: for each speaker, we selected the 10 sentences that were ranked most highly to count as high arousing stimuli, and 10 sentences that were ranked lowest as low arousing stimuli. Thus, for each emotional category, 20 sentences were categorized as low and 20 sentences were categorized as high arousing stimuli. The 10 sentences rated as “medium” arousal were not included in the ERP analysis.

**Table 1:** **Acoustic measures of emotional expressions produced by both speakers**.

**Emotion**	**Mean *F*_0_ (Hz)**	**Range *F*_0_ (Hz)**	**Mean dB**	**Range dB**	**Duration (s)**
Anger	264.4	219.8	71.2	50.0	2.9
Disgust	196.2	234.5	69.7	45.5	3.6
Fear	195.7	225.3	68.3	42.0	3.7
Sadness	184.6	210.8	69.2	44.9	3.1
Happiness	267.6	310.8	70.8	49.8	2.9
Surprise	316.6	312.3	70.9	52.2	3.0

### Procedure

After preparation for EEG recordings, participants were seated in an electrically shielded chamber at a distance of approx. 115 cm in front of a monitor. Auditory stimuli were presented via loudspeakers positioned directly to the left and right side of the monitor. Stimuli were pseudo-randomized and presented to the participant split into 10 blocks of 72 trials each. Half of the participants carried out an “implicit task” (“How aroused do you feel when listening to the sentence”) and the other half carried out an “explicit task” (How aroused did the speaker feel when uttering the sentence?”). Task distribution was counterbalanced across participants. A trial was as follows: before the onset of each auditory stimulus, a fixation cross was presented in the center of the screen for 200 ms. This was immediately followed by the stimulus presentation (sentence duration was max. 3000 ms long). Following this, a nine-point arousal scale appeared on the screen for 200 ms, prompting the participant to respond. After the response, an inter-stimulus interval (ISI) of 1500 ms followed, before the next stimulus was presented. After each block, the participant paused for a self-determined duration before proceeding.

### ERP recording

The EEG was recorded from 49 Ag–AgCl electrodes mounted on a custom-made cap (Electro-Cap International) according to the modified expanded 10–20 system (Nomenclature of the American Electroencephalographic Society, 1991). Signals were recorded continuously with a band pass between DC and 70 Hz and digitized at a sampling rate of 500 Hz (Xrefa amplifier). The reference electrode was placed on the left mastoid. Bipolar horizontal and vertical EOGs were recorded for artifact rejection purposes. Electrode resistance was kept below 5 KΩ. Data was re-referenced offline to linked mastoids. The data was inspected visually in order to exclude trials containing extreme artifacts and drifts, and all trials containing EOG-artifacts above 30.00 μV were rejected automatically. In total, approximately 16% of the data was rejected. Trials were averaged over a time range of 200 ms before stimulus onset to 1000 ms after stimulus onset.

### Data analysis

For the ERP analysis, the electrodes were grouped according to regions of interests. Left frontal electrode-sites: F5, F3, FC5, FC3; left central sites: C5, C3, CP5, CP3; left posterior sites: P5, P3, PO7, PO3; right frontal sites: F6, F4, FC6, FC4; right central sites: C6, C4, CP6, CP4; and right posterior sites: P6, P4, PO8, PO4. Based on visual inspection and previous evidence, an early time-window from 170 to 230 ms (P200 component, Paulmann et al., 2010) and a later time-window from 450 to 750 ms after sentence onset (LPC component, Lazlo and Federmeier, 2009) were selected for analysis of mean amplitudes.

Mean amplitudes were entered into a repeated measurements ANOVA using the within-subject factors arousal (high, low), emotion (anger, disgust, fear, sadness, happiness, pleasant surprise), region of interest [six ROIs: left/right frontal (LF), left/right central (LC), left/right posterior (LP) electrode-sites], and the between-subjects factor task (implicit/explicit stimulus evaluation). Customized tests of hypotheses *(post hoc* tests) were carried out using a modified Bonferroni procedure correction for multiple comparisons when appropriate (see Keppel, 1991). Therefore, in cases where all emotions were contrasted with one another (15 contrasts in total), the alpha level for significance testing was set at *p* < 0.017 and not at *p* < 0.05. Comparisons with more than one degree of freedom in the numerator were corrected for non-sphericity using the Greenhouse–Geisser correction (Greenhouse and Geisser, 1959). The graphs displayed were filtered with a 7 Hz low-pass filter.

## Results

For the ease of reading, only significant main effects and interactions involving the critical factors *emotion, arousal*, and/or *task* are reported.

### P200 mean amplitudes (170–230 ms)

In the early time-window a significant effect of *emotion* [*F*(5, 185) = 3.25, *p* = 0.01] was found, revealing differently modulated amplitudes for sentences spoken in the different tones of voice. This main effect was qualified by a significant two-way interaction between *emotion* and *ROI* [*F*(25, 925) = 2.38, *p* = 0.01]. *Post hoc* contrasts at each ROI revealed the following patterns. At LF electrode-sites, sentences intoned in an angry tone differed significantly from sentences intoned in a fearful voice [*F*(1, 37) = 10.21, *p* < 0.01], as well as in a sad tone [*F*(1, 37) = 22.43, *p* < 0.0001]. At this ROI, there was also a marginal difference between pleasant surprise and sad sentences [*F*(1, 37) = 4.42, *p* < 0.05]. At LM sites, angry sentences could again be distinguished from fearful [*F*(1, 37) = 10.38, *p* < 0.01] and sad [*F*(1, 37) = 4.42, *p* = 0.019] sentences. Additionally, ERPs for disgust sentences differed marginally from fearful sentences [*F*(1, 37) = 4.82, *p* < 0.05]. Fearful sentences also differed significantly from happy [*F*(1, 37) = 8.38, *p* < 0.01] and marginally from pleasant surprise sentences [*F*(1, 37) = 4.33, *p* < 0.05]. ERPs in response to happy and sad sentences also differed marginally [*F*(1, 37) = 4.54, *p* < 0.05] at LM sites, as well as at LP sites [*F*(1, 37) = 4.45, *p* < 0.05]. At RF sites, ERPs in response to angry sentences differed from ERPs in response to fearful [*F*(1, 37) = 10.37, *p* < 0.01] and sad [*F*(1, 37) = 19.71, *p* < 0.0001] sentences. ERPs to fearful sentences also differed from pleasant surprise [*F*(1, 37) = 8.53, *p* < 0.01] and marginally from happy [*F*(1, 37) = 5.02, *p* = 0.03] sentences. The same was found for the contrasts between ERPs in response to sad sentences and pleasant surprise [*F*(1, 37) = 11.71, *p* < 0.01] and sad and happy [*F*(1, 37) = 6.13, *p* = 0.018] sentences. At RM sites, a similar pattern emerged: ERPs in response to angry sentences differed significantly from fearful [*F*(1, 37) = 10.90, *p* < 0.01] and sad sentences [*F*(1, 37) = 17.10, *p* < 0.001], and marginally from disgust sentences [*F*(1, 37) = 4.33, *p* < 0.05]. Moreover, ERPs in response to disgust and happy sentences differed [*F*(1, 37) = 6.35, *p* < 0.017] as did ERPs in response to fearful and happy [*F*(1, 37) = 7.58, *p* < 0.01] and pleasant surprise [*F*(1, 37) = 8.08, *p* < 0.01]. ERPs in response to sad sentences differed significantly from happy [*F*(1, 37) = 7.83, *p* < 0.01] and pleasant surprise [*F*(1, 37) = 9.13, *p* < 0.01] sentences. No significant differences were found at RP sites.

**Figure 1:**
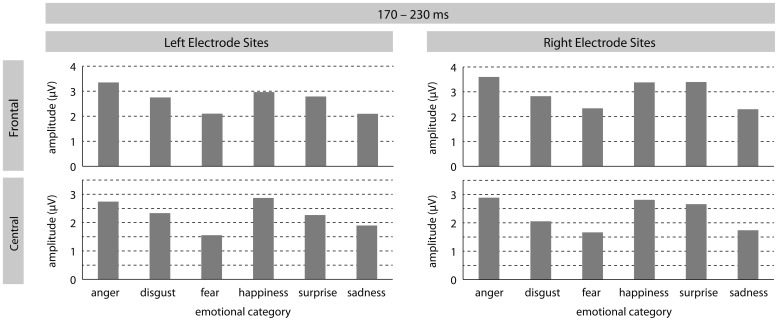
**The illustration shows mean P200 amplitudes (in mV) for each emotional category at left/right frontal and left/right central electrode-sites**.

Finally, there was also a marginally significant main effect of *arousal* [*F*(1, 37) = 3.28, *p* = 0.078], revealing a stronger positivity for high arousing stimuli when compared to low arousing stimuli. No other main effects or interactions turned out to be significant. See Figures 1 and 3 for visualization of effects.

In summary, data analysis confirms a significant *emotion* effect revealing early differentiation of vocal emotional expressions in the P200 amplitude though individual contrasts between specific emotional tones seem to vary as a function of distribution. Significant differentiation effects are primarily found at frontal and central electrode-sites. Also, the analysis revealed a marginally significant effect of *arousal* with high arousing stimuli eliciting more positive P200 amplitudes than low arousing stimuli. Finally, there was no indication that task instructions influenced P200 amplitude modulation.

### LPC mean amplitudes (450–750 ms)

In the later time-window, a significant effect of *emotion* was found [*F*(5, 185) = 7.22, *p* < 0.0001], revealing differently modulated LPC amplitudes for the different emotional sentences. All *post hoc* contrasts comparing each emotion with one another turned out to be significant (all *F*'s > 6.5; all *p*'s < 0.017). The main effect of *emotion* also interacted with *ROI* [*F*(25, 925) = 4.48, *p* < 0.0001] suggesting distribution differences for the *emotion* effect. *Post hoc* contrasts at LM sites revealed significant differences between ERPs in response to disgust and angry [F (1, 37) = 13.08, *p* < 0.001], fearful [*F*(1, 37) = 6.91, *p* < 0.017], happy [*F*(1, 37) = 12.58, *p* < 0.001], and pleasant surprise [*F*(1, 37) = 13.66, *p* < 0.001] sentences. At this ROI, the contrast between happy and sad sentences also turned out to be marginally significant [*F*(1, 37) = 4.68, *p* < 0.05]. At LP sites, the following contrasts reached significance: anger vs. disgust [*F*(1, 37) = 31.08, *p* < 0.0001]; anger vs. fear [*F*(1, 37) = 12.69, *p* < 0.001]; anger vs. sadness [*F*(1, 37) = 22.32, *p* < 0.0001]; disgust vs. happiness [*F*(1, 37) = 17.10, *p* < 0.001]; disgust vs. pleasant surprise [*F*(1, 37) = 26.24, *p* < 0.0001];fear vs. happiness [*F*(1, 37) = 9.03, *p* < 0.01]; fear vs. pleasant surprise [*F*(1, 37) = 14.30, *p* < 0.001]; happy vs. sadness [*F*(1, 37) = 38.29, *p* < 0.0001]; and pleasant surprise vs. sadness [*F*(1, 37) = 23.14, *p* < 0.0001]. At RF electrode-sites, ERPs in response to disgust sentences differed significantly from fearful [*F*(1, 37) = 13.10, *p* < 0.001], happy [*F*(1, 37) = 10.89, *p* < 0.01], and pleasant surprise [*F*(1, 37) = 7.69, *p* < 0.01] sentences. The contrasts between disgust and sad sentences almost reached significance [*F*(1, 37) = 4.20, *p* < 0.05], as did the contrast between happy and sad sentences [*F*(1, 37) = 5.65, *p* < 0.03]. At RM electrode-sites, results revealed a (marginal) significant difference between LPC amplitudes for angry and disgust [*F*(1, 37) = 19.37, *p* < 0.0001] and angry and sad [*F*(1, 37) = 5.50, *p* < 0.03] sentences. Disgust sentences were found to differ from all other emotional sentences except for sad stimuli (all *F*'s > 17.0 and all *p*'s < 0.001). LPCs for sad and happy sentences also differed [*F*(1, 37) = 19.26, *p* < 0.0001] as did sad and pleasant surprise sentences [*F*(1, 37) = 7.12, *p* < 0.01]. Finally, at RP sites, ERPs to angry sentences differed from ERPs to disgust [*F*(1, 37) = 31.56, *p* < 0.0001], fearful [*F*(1, 37) = 5.90, *p* = 0.02], and sad [*F*(1, 37) = 14.78, *p* < 0.001] sentences. Similar to RM sites, disgust sentences were again found to differ from all other emotional sentences except for sad sentences (all *F*'s > 10.36 and all *p*'s < 0.001). In addition, ERPs in response to fearful sentences were significantly different from ERPs in response to pleasant surprise [*F*(1, 37) = 8.45, *p* < 0.001] and marginally different from ERPs in response to happy [*F*(1, 37) = 8.45, *p* = 0.02] sentences. Comparable to LP sites, sad sentences also elicited different LPC amplitudes to happy [*F*(1, 37) = 26.47, *p* < 0.0001] and pleasant surprise [*F*(1, 37) = 15.32, *p* < 0.001] sentences at RP sites.

**Figure 2:**
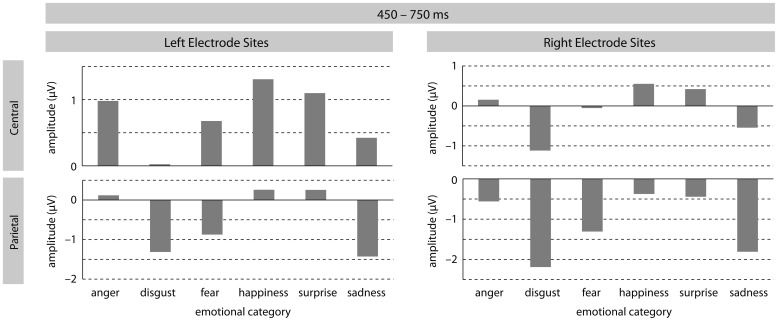
**The illustration shows mean LPC amplitudes (in mV) for each emotional category at left/right central and left/right parietal electrode-sites**.

**Figure 3:**
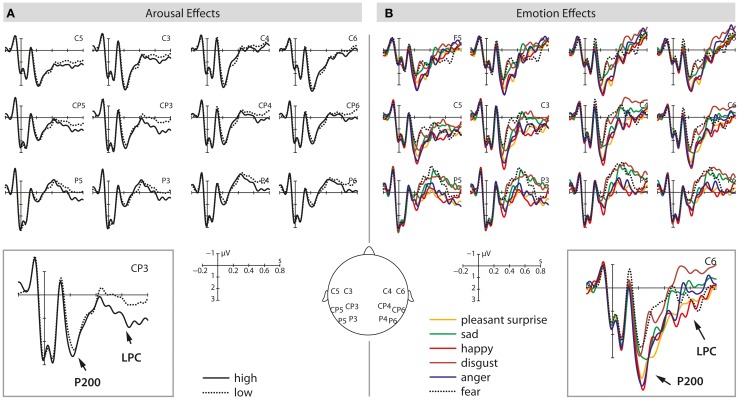
**This illustration shows the P200 and LPC effects at selected electrodes for high/low arousing stimuli (A) and the six different emotional categories tested (B)**. The left panel shows average waveforms for high (solid) and low (dotted) arousing stimuli from 200 ms before stimulus onset up to 800 ms after stimulus onset. On the right side, average waveforms for different emotional prosodies are displayed from 200 ms before sentence onset up to 800 ms after stimulus onset.

The analysis also revealed a marginally significant main effect of *arousal* [*F*(1, 37) = 3.29, *p* = 0.08] as well as a significant interaction between *arousal* and *ROI* [*F*(5, 185) = 3.20, *p* < 0.05]. Arousal effects were significant at LM, LP, and RP ROIs (all *F*'s > 4.94 and all *p*'s < 0.05). In all instances, high arousing stimuli elicited more positive-going amplitudes than low arousing stimuli. Last, there was a significant three-way interaction *emotion* × *arousal* × *ROI* [*F*(25, 925) = 1.98, *p* < 0.05] but step-down analyses by *ROI* revealed no further significant effects. See Figures 2 and 3 for visualization of effects.

In sum, analyses for LPC amplitudes revealed that different emotional prosodies can be distinguished from one another in this late time-window. In addition, arousal effects turned out to be significant. Again, there was no indication that task instructions influence this differentiation in the present data.

## Discussion

To the best of our knowledge, this is the first ERP study simultaneously investigating the temporal dynamics of emotion and arousal effects on early (P200) and late (LPC) ERP components when processing affective information from prosodic speech materials. We report an early differentiation of six basic emotions as reflected in differently modulated P200 amplitudes at fronto-central electrode-sites. In addition, high arousing stimuli elicited slightly stronger P200 amplitudes than low arousing stimuli. The P200 effect was followed by an LPC in which the different emotions could again be differentiated from each other. Also, high arousing stimuli elicited larger LPCs than low arousing stimuli. No interaction between the two factors nor an influence of task focus was found in either time-window. Taken together, the results are thus in line with reports from visual affective language and picture processing which suggest that emotion or valence relevant information is extracted before arousal relevant information (e.g., Keil et al., 2002; Gianotti et al., 2008). Below, we will outline how the current results contribute to our understanding of affective prosody processing.

### P200

Differently modulated P200 amplitudes in response to emotional speech materials have been repeatedly reported in the literature (e.g., Paulmann and Kotz, 2008; Paulmann et al., 2010; Schirmer et al., 2013; Garrido-Vásquez et al., in press). However, previously, authors only tested whether emotional materials could be differentiated from neutral materials. Here, we extend these findings by reporting that different emotional categories can also be distinguished from one another in this early time-window. This goes in line with an earlier tentative suggestion that specific emotional categories can be inferred from rather short stimulus durations (e.g., Paulmann and Pell, 2010), i.e., within 200 ms of stimulus onset. We have previously theorized that early emotional detection as reflected in the P200 is primarily based on the integration of emotionally relevant salient acoustic features including pitch, tempo, voice quality, and loudness. Some authors have claimed that the sensitivity of the P200 to physical stimulus attributes undermines the interpretation that it can reflect early emotional decoding (see Schirmer et al., 2013). However, given that stimuli with a similar acoustic profile (e.g., fear and disgust, see Table 1) can still be differentiated in the P200 makes this criticism less severe. In fact, more systematic P200 variations should be found if the early effect was only driven by a single acoustic parameter (i.e., stimuli with the same intensity or same pitch should elicit non-differentiable P200 amplitudes). Moreover, there is evidence by Stekelenburg and Vroomen (2007, 2012) which shows dissociations between N1 effects that were linked to processing general visual/auditory physical stimulus characteristic and P2 effects which were linked to processing of phonetic, semantic, or associative information. Taken together, it thus seems reasonable to suggest that P200 amplitude differences reflect emotional salience detection rather than sensory processing only. Crucially, researchers exploring emotional prosody perception have previously argued that matching acoustical attributes across stimuli from different categories would result in a serious reduction of the emotionality conveyed by a specific stimulus (e.g., Wiethoff et al., 2008) given that emotions are conveyed through a specific combination of different acoustic features (e.g., Banse and Scherer, 1996; Paulmann et al., 2008a). Artificially changing or removing these features results in ecologically less valid stimuli. Finally, in the neuro-imaging literature, some authors (e.g., Alba-Ferrara et al., 2011) have tried to statistically control for the influence of primary acoustic features such as pitch. Generally, similar brain activation patterns were found for stimuli that differed with regard to specific acoustical features (e.g., pitch), once more suggesting that emotional prosody evaluation is not driven by a single parameter. Rather, specific acoustic configuration patterns seem to convey emotionality through the voice. Future research should thus aim to explore which combination of acoustic parameters drives early emotional evaluation.

The present findings also revealed a marginally significant P200 effect of arousal irrespective of the emotional category tested. While most previous studies report late arousal effects for visually presented emotional materials (e.g., Herbert et al., 2006; Schupp et al., 2007; Hinojosa et al., 2009), there are some indications that arousal information can be extracted at an early stage of emotional processing, too (Hofmann et al., 2009; Feng et al., 2012). For instance, Hofmann and colleagues report an early EPN effect of arousal when processing negative (but not positive) word stimuli while participants carried out a lexical decision task suggesting that arousal characteristics can facilitate lexical processing. Feng et al. (2012) describe that the P2b component was influenced by arousal in their implicit picture viewing task (participants had to identify the color of the picture frame). High arousing pictures elicited larger P2 amplitudes at posterior electrode-sites than low arousing pictures. The discrepancy between studies reporting only late arousal effects and those reporting early arousal effects is often linked to task differences. Both Hofmann et al. (2009) and Feng et al. (2012) used tasks in which participants were not required to focus on emotional *or* arousal attributes while both of our task instructions focused on arousal attributes. Future studies should thus explore whether early effects only robustly arise if emotional or arousal evaluation is not in task focus.

Alternatively, discrepancies in the literature with regard to the temporal dynamics of effects could result from differences in stimulus duration. Early arousal effects for non-language stimuli have usually been reported for stimuli that were only *briefly* presented (e.g., 300 ms in Feng et al., 2012, or 120 ms in Schupp et al., 2004). The explanation seems less likely to apply to language-relevant stimuli though given that early arousal effects are reported for words that were presented for 1000 ms (Hofmann et al., 2009). Together with the marginal effect reported here, this suggests that for language stimuli, stimulus duration is not crucially influencing arousal effects.

In sum, the current findings suggest that emotionality detection seems to be more relevant to listeners than extraction of arousal information at an early processing stage. However, arousal characteristics of stimuli do not seem to go completely unnoticed. We thus theorize that the P200 is robustly modulated by emotional significance of an affective prosodic stimulus independent of task focus as no task differences were found in the present or in previous studies. Though less robust, the P200 can also be modulated by arousal features of stimuli suggesting that arousal level of speakers can impact on the way they produce emotional prosody. Hence, we propose that the P200 reflects early facilitated processing of motivationally or emotionally relevant stimuli. These intrinsic relevant features are transmitted through a combination of different acoustic parameters thus leaving open the possibility that part of this early emotional detection mechanism is influenced by sensory processing.

### Late positive complex

Next to assessing whether arousal and emotionality of stimuli can influence early processing mechanisms, the present study also set out to investigate in how far the later LPC can be influenced by these two factors. Results showed that all emotional expressions elicited differently modulated LPCs at central-posterior electrode-sites (bilaterally). In addition, LPC amplitudes were differently modulated for high as opposed to low arousing stimuli irrespective of which emotion they belonged to. This effect was slightly more prominent at left centro-parietal electrodes than at their right lateralized counter parts. No influence of task focus was observed in the present study and we also fail to find an interaction between emotion and arousal attributes of stimuli.

The finding that different emotions elicit differently modulated and differently distributed LPC amplitudes fits well with observations from the imaging literature on emotional language processing, which revealed a diversified bilateral brain network of cortical and sub-cortical brain structures underlying emotion processing in speech (e.g., Kotz et al., 2003; Grandjean et al., 2005; Wildgruber et al., 2005; Ethofer et al., 2009; and see e.g., Kotz and Paulmann, 2011 for review). Moreover, imaging studies that explored both arousal and valence, seem to suggest that two distinct neural systems underlie the processing of these two dimensions. In these studies, arousal processing has predominantly been linked to sub-cortical brain structures (e.g., amygdala) while emotion processing has been linked to frontal cortex activity (e.g., Lewis et al., 2007). The present data show a similar neural dissociation as distribution of arousal effects clearly differed from the distribution of emotion effects (e.g., the arousal effect was primarily visible over left hemisphere electrode-sites). It thus seems sensible to suggest that emotion and arousal processing relies at least partially on differing neural mechanisms. However, given that ERPs lack the accurate spatial resolution of other imaging techniques this interpretation of distribution differences remains tentative.

As for a functional interpretation of the LPC, previous visual emotion studies have linked the component to reflect enhanced or continuous analysis of emotionally relevant visual stimuli (e.g., Cuthbert et al., 2000; Kanske and Kotz, 2007; Hinojosa et al., 2009; Bayer et al., 2010; Leite et al., 2012). Here, we propose to extend this interpretation to stimuli that convey emotionality or arousal only through the tone of voice that they are uttered in. In line with multi-step processing models of affective prosody (e.g., Schirmer and Kotz, 2006; Kotz and Paulmann, 2011), the present findings confirm that early emotional salience detection is followed by more elaborate processing of stimuli. Specifically, we suggest that larger LPC effects for high arousing stimuli reflect persevere processing of salient affective information which might ultimately lead to preferential processing of emotionally relevant stimuli similar to reports of other previously observed later ERP components (e.g., late negativity in Paulmann et al., 2011). In a recent emotional prosody processing study, Schirmer et al. (2013) present findings that modulations in the early P200 component can predict evaluation differences in the subsequently observed LPC component. While the direct influence of the P200 on the concurrent LPC was not directly tested here, it seems reasonable to assume that stimuli which have been identified as potentially relevant (e.g., due to their emotionality or arousal level), need to be thoroughly processed and analyzed to ensure appropriate subsequent social behavior (e.g., fight/flight). While arousal effects were only marginally significant in the P200 component, the LPC seems to be robustly modulated by both the arousal and emotion dimension though no interaction between the two factors was observed (c.f., Leite et al., 2012 for similar finding when participants had to view pictures). That is, the present findings are in line with the view that the LPC might simply reflect enhanced processing of stimuli that carry potentially relevant affective information (e.g., Cuthbert et al., 2000; Bayer et al., 2010; Leite et al., 2012). This processing step seems to be unrelated to arousal level of participants (i.e., how much they potentially engage with the stimulus) as we find significant LPC effects under both task instructions tested (c.f., Bayer et al., 2010 for similar interpretation of LPC effects for visual sentence processing).

### The influence of task instructions on the P200 and LPC

The present experimental design also allowed testing for the influence of task instructions on the P200 and LPC component. In the “implicit” task condition, participants were asked to evaluate their own arousal level after listening to the stimulus, while in the “explicit” task condition they were required to evaluate the arousal level of the speaker. Thus, the only difference between the two tasks was level of attention that participants had to pay to our stimuli. No influence of task instructions was observed suggesting that both early as well as subsequent more enhanced affective analyses are largely independent of task focus of participants. The lack of task influence for early emotional decoding (P200 component) has previously been documented (Garrido-Vásquez et al., in press). Here, we extend previous findings by reporting evidence which suggests that a possible early evaluation of arousal attributes is also not dependent on task instructions. Hence, the P200 component seems to be robustly elicited irrespective of how much participants need to attend to the stimulus.

In contrast, the LPC is reported to be more vulnerable to task demands. For instance, Schacht and Sommer (2009) report enhanced LPC amplitudes to emotional words only when participants engaged in lexical or semantic task evaluations, but not when participants had to report whether they saw an italicized letter (structural task). Here, participants had to attend to the affective attributes of stimuli in some way which could explain why LPC amplitudes did not differ between our two tasks. Future studies will have to shed further light on the impact task effects can have on the LPC when task foci are very different. For now, we propose that the LPC in response to affective auditory stimuli is not heavily influenced by task focus for as long as participants pay at least some attention to the affective properties of stimuli. This idea is in line with results from the visual emotion literature (e.g., Feng et al., 2012) showing that emotion and arousal can affect affective processing stages even when participants engage in implicit tasks and do not have a “task-related motivation” to analyze stimuli.

## Conclusion

This study set out to explore the influence of emotion and arousal on early and later ERP components. In line with findings from the literature on visual emotion processing, our results suggest that emotion relevant details are robustly decoded during early (P200) and late processing (LPC) stages while arousal information is only reliably taken into consideration at later stages of processing. Given the lack of an interaction between the two factors of interest, the results also suggest that the two dimensions are largely independent of each other (c.f., Russell, 1980) at least when stimuli are attended to and somewhat task-relevant.

### Conflict of interest statement

The authors declare that the research was conducted in the absence of any commercial or financial relationships that could be construed as a potential conflict of interest.

## References

[B1] Alba-Ferrara L. Hausmann M. Mitchell R. L. Weis S. (2011). The neural correlates of emotional prosody comprehension: disentangling simple from complex emotion. PLoS ONE 6:e28701 10.1371/journal.pone.0028701 22174872 PMC3236212

[B2] American Electroencephalographic Society. (1991). Guidelines for standard electrode position nomenclature. J. Clin. Neurophysiol. 8, 200–202 10.1097/00004691-199104000-00007 2050819

[B3] Banse R. Scherer K. R. (1996). Acoustic profiles in vocal emotion expression. J. Pers. Soc. Psychol. 3, 614–636 10.1037/0022-3514.70.3.614 8851745

[B4] Bayer M. Sommer W. Schacht A. (2010). Reading emotional words within sentences: the impact of arousal and valence on event-related potentials. Int. J. Psychophysiol. 78, 299–307 10.1016/j.ijpsycho.2010.09.004 20854848

[B5] Bostanov V. Kotchoubey B. (2004). Recognition of affective prosody: continuous wavelet measures of event-related brain potentials to emotional exclamations. Psychophysiology 41, 259–268 10.1111/j.1469-8986.2003.00142.x 15032991

[B6] Cuthbert B. N. Schupp H. T. Bradley M. M. Birbaumer N. Lang P. J. (2000). Brain potentials in affective picture processing: covariation with autonomic arousal and affective report. Biol. Psychol. 52, 95–111 10.1016/S0301-0511(99)00044-7 10699350

[B6a] Ethofer T. Kreifelts B. Wiethoff S. Wolf J. Grodd W. Vuilleumier P. (2009). Differential influences of emotion, task, and novelty on brain regions underlying the processing of speech melody. J. Cogn. Neurosci. 21, 1255–1268 10.1162/jocn.2009.21099 18752404

[B7] Feldman-Barrett L. (2006). Solving the emotion paradox: categorization and the experience of emotion. Pers. Soc. Psychol. Rev. 10, 20–46 10.1207/s15327957pspr1001_2 16430327

[B8] Feldman-Barrett L. Mesquita B. Ochsner K. N. Gross J. J. (2007). The experience of emotion. Annu. Rev. Psychol. 58, 373 10.1146/annurev.psych.58.110405.085709 17002554 PMC1934613

[B9] Feldman-Barrett L. Russell J. A. (1998). Independence and bipolarity in the structure of current affect. J. Pers. Soc. Psychol. 74, 967–984 10.1037/0022-3514.74.4.967

[B10] Feng C. Wang L. Liu C. Zhu X. Dai R. Mai X. (2012). The time course of the influence of valence and arousal on the implicit processing of affective pictures. PLoS ONE 7:e29668 10.1371/journal.pone.0029668 22295062 PMC3266261

[B11] Garrido-Vásquez P. Pell M. D. Paulmann S. Strecker K. Schwarz J. Kotz S. A. (in press). An ERP study of vocal emotion processing in asymmetric Parkinson's disease. Soc. Cogn. Affect. Neurosci. 10.1093/scan/nss094 22956665 PMC3831560

[B12] Gianotti L. R. Faber P. L. Schuler M. Pascual-Marqui R. D. Kochi K. Lehmann D. (2008). First valence, then arousal: the temporal dynamics of brain electric activity evoked by emotional stimuli. Brain Topogr. 20, 143–156 10.1007/s10548-007-0041-2 18175212

[B13] Grandjean D. Sander D. Pourtois G. Schwartz S. Seghier M. L. Scherer K. R. (2005). The voices of wrath: brain responses to angry prosody in meaningless speech. Nat. Neurosci. 8, 145–146 10.1038/nn1392 15665880

[B14] Greenhouse S. Geisser S. (1959). On methods in the analysis of profile data. Psychometrika 24, 95–112 10.1007/BF02289823

[B15] Herbert C. Kissler J. Junghöfer M. Peyk P. Rockstroh B. (2006). Processing of emotional adjectives: evidence from startle EMG and ERPs. Psychophysiology 43, 197–206 10.1111/j.1469-8986.2006.00385.x 16712590

[B16] Hinojosa J. A. Carretié L. Méndez-Bértolo C. Míguez A. Pozo M. A. (2009). Arousal contributions to affective priming: electrophysiological correlates. Emotion 9, 164–171 10.1037/a0014680 19348529

[B17] Hofmann M. J. Kuchinke L. Tamm S. Võ M. L. Jacobs A. M. (2009). Affective processing within 1/10th of a second: high arousal is necessary for early facilitative processing of negative but not positive words. Cogn. Affect. Behav. Neurosci. 9, 389–397 10.3758/9.4.389 19897792

[B18] Kanske P. Kotz S. A. (2007). Concreteness in emotional words: ERP evidence from a hemifield study. Brain Res. 1148, 138–148 10.1016/j.brainres.2007.02.044 17391654

[B19] Keil A. Bradley M. M. Hauk O. Rock-stroh B. Elbert T. Lang P. J. (2002). Large-scale neural correlates of affective picture processing. Psychophysiology 39, 641–649 10.1111/1469-8986.3950641 12236331

[B20] Keppel G. (1991). Design and Analysis: A Researcher's Handbook. Englewood Cliffs, NJ: Prentice Hall

[B21] Kotz S. A. Meyer M. Alter K. Besson M. von Cramon D. Y. Friederici A. D. (2003). On the lateralization of emotional prosody: an event-related functional MR investigation. Brain Lang. 86, 366–376 10.1016/S0093-934X(02)00532-1 12972367

[B22] Kotz S. A. Paulmann S. (2007). When emotional prosody and semantics dance cheek to cheek: ERP evidence. Brain Res. 1151, 107–118 10.1016/j.brainres.2007.03.015 17445783

[B23] Kotz S. A. Paulmann S. (2011). Emotion, language and the brain. Lang. Linguist. Compass 5, 108–125 10.1111/j.1749-818X.2010.00267.x

[B24] Lazlo S. Federmeier K. (2009). A beautiful day in the neighborhood: an event-related potential study of lexical relationships and prediction in context. J. Mem. Lang. 61, 326–338 10.1016/j.jml.2009.06.004 20161064 PMC2747758

[B25] Leite J. Carvalho S. Galdo-Alvarez S. Alves J. Sampaio A. Gonçalves, Ó F. (2012). Affective picture modulation: valence, arousal, attention allocation and motivational significance. Int. J. Psychophysiol. 83, 375–381 10.1016/j.ijpsycho.2011.12.005 22226675

[B26] Lewis P. A. Critchley H. D. Rotshtein P. Dolan R. J. (2007). Neural correlates of processing valence and arousal in affective words. Cereb. Cortex 17, 742–748 10.1093/cercor/bhk024 16699082 PMC2267931

[B27] Olofsson J. K. Nordin S. Sequeira H. Polich J. (2008). Affective picture processing: an integrative review of event-related potential findings. Biol. Psychol. 77, 247–265 10.1016/j.biopsycho.2007.11.006 18164800 PMC2443061

[B28] Paulmann S. Kotz S. A. (2008). Early emotional prosody perception based on different speaker voices. Neuroreport 19, 209–213 10.1097/WNR.0b013e3282f454db 18185110

[B29] Paulmann S. Ott D. V. Kotz S. A. (2011). Emotional speech perception unfolding in time: the role of the basal ganglia. PLoS ONE 6:e17694 10.1371/journal.pone.0017694 21437277 PMC3060083

[B30] Paulmann S. Pell M. D. (2010). Contextual influences of emotional speech prosody on face processing: how much is enough? Cogn. Affect. Behav. Neurosci. 10, 230–242 10.3758/CABN.10.2.230 20498347

[B31] Paulmann S. Pell M. D. Kotz S. A. (2008a). How aging affects the recognition of emotional speech. Brain Lang. 104, 262–269 10.1016/j.bandl.2007.03.002 17428529

[B32] Paulmann S. Schmidt P. Pell M. D. Kotz S. A. (2008b). Rapid processing of emotional and voice information as evidenced by ERPs, in Proceedings of the Conference on Speech Prosody 2008, eds Barbosa P. A. Madureira S. Reis C. (Campinas), 205–209

[B33] Paulmann S. Seifert S. Kotz S. A. (2010). Orbito-frontal lesions cause impairment during late but not early emotional prosodic processing. Soc. Neurosci. 5, 59–75 10.1080/17470910903135668 19658025

[B34] Pell M. D. Monetta L. Paulmann S. Kotz S. A. (2009). Recognizing emotions in a foreign language. J. Nonverbal Behav. 33, 107–120 10.1007/s10919-008-0065-7

[B35] Pihan H. Altenmüller E. Ackermann H. (1997). The cortical processing of perceived emotion: a DC-potential study on affective speech prosody. Neuroreport 8, 623–627 10.1097/00001756-199702100-00009 9106735

[B36] Russell J. A. (1980). A circum-plex model of affect. J. Pers. Soc. Psychol. 39, 1161–1178 10.1037/h0077714

[B37] Schacht A. Sommer W. (2009). Time course and task dependence of emotion effects in word processing. Cogn. Affect. Behav. Neurosci. 9, 28–43 10.3758/CABN.9.1.28 19246325

[B38] Schirmer A. Chen C. B. Ching A. Tan L. Hong R. Y. (2013). Vocal emotions influence verbal memory: neural correlates and interindividual differences. Cogn. Affect. Behav. Neurosci. 13, 80–93 10.3758/s13415-012-0132-8 23224782

[B39] Schirmer A. Kotz S. A. (2003). ERP evidence for a sex-specific Stroop effect in emotional speech. J. Cogn. Neurosci. 15, 1135–1148 10.1162/089892903322598102 14709232

[B40] Schirmer A. Kotz S. A. (2006). Beyond the right hemisphere: brain mechanisms mediating vocal emotional processing. Trends Cogn. Sci. (Regul. Ed.) 10, 24–30 10.1016/j.tics.2005.11.009 16321562

[B41] Schirmer A. Kotz S. A. Friederici A. D. (2002). Sex differentiates the role of emotional prosody during word processing. Cogn. Brain Res. 14, 228–233 10.1016/S0926-6410(02)00108-8 12067695

[B42] Schirmer A. Striano T. Friederici A. D. (2005a). Sex differences in the preattentive processing of vocal emotional expressions. Neuroreport 16, 635–639 10.1097/00001756-200504250-00024 15812323

[B43] Schirmer A. Kotz S. A. Friederici A. D. (2005b). On the role of attention for the processing of emotions in speech: sex differences revisited. Cogn. Brain Res. 24, 442–452 10.1016/j.cogbrainres.2005.02.022 16099357

[B44] Schupp H. T. Junghöfer M. Weike A. I. Hamm A. O. (2004). The selective processing of briefly presented affective pictures: an ERP analysis. Psychophysiology 41, 441–449 10.1111/j.1469-8986.2004.00174.x 15102130

[B45] Schupp H. T. Stockburger J. Codispoti M. Junghöfer M. Weike A. I. Hamm A. O. (2007). Selective visual attention to emotion. J. Neurosci. 27, 1082–1089 10.1523/JNEUROSCI.3223-06.2007 17267562 PMC6673176

[B46] Stekelenburg J. J. Vroomen J. (2007). Neural correlates of multisensory integration of ecologically valid audiovisual events. J. Cogn. Neurosci. 19, 1964–1973 10.1162/jocn.2007.19.12.1964 17892381

[B47] Stekelenburg J. J. Vroomen J. (2012). Electrophysiological correlates of predictive coding of auditory location in the perception of natural audiovisual events. Front. Integr. Neurosci. 6:26 10.3389/fnint.2012.00026 22666195 PMC3364694

[B48] Wambacq I. J. Shea-Miller K. J. Abubakr A. (2004). Nonvoluntary and voluntary processing of emotional prosody: an event-related potentials study. Neuroreport 15, 555–559 10.1097/00001756-200403010-00034 15094522

[B49] Wambacq I. J. A. Jerger J. F. (2004). Processing of affective prosody and lexical-semantics in spoken utterances as differentiated by event-related potentials. Cogn. Brain Res. 20, 427–437 10.1016/j.cogbrainres.2004.03.015 15268920

[B50] Wiethoff S. Wildgruber D. Becker H. Anders S. Herbert C. Grodd W. (2008). Cerebral processing of emotional prosody – influence of acoustic parameters and arousal. Neuroimage 39, 885–893 10.1016/j.neuroimage.2007.09.028 17964813

[B51] Wildgruber D. Riecker A. Hertrich I. Erb M. Grodd W. Ethofer T. (2005). Identification of emotional intonation evaluated by fMRI. Neuroimage 24, 1233–1241 10.1016/j.neuroimage.2004.10.034 15670701

